# Safety and effectivity of Kono-S anastomosis in Crohn’s patients: a systematic review and Meta-analysis

**DOI:** 10.1007/s00423-024-03412-x

**Published:** 2024-07-22

**Authors:** Marionna Cathomas, Baraa Saad, Stephanie Taha-Mehlitz, Dilip K. Vankayalapati, Nour El Ghazal, Mohammed Majd Mourad, Niklas Ortlieb, Christian A. Than, Emanuel Burri, Christine Glaser, Andres Heigl, Katerina Neumann, Michael D. Honaker, Anas Taha, Robert Rosenberg

**Affiliations:** 1 Department of Surgery, Cantonal Hospital Baselland, Rheinstrasse 26, Liestal, 4410 Switzerland; 2 School of Medicine, St George’s University of London, London, SW17 0RE UK; 3 Clarunis, Department of Visceral Surgery, University Center for Gastrointestinal and Liver Diseases, St. Clara Hospital and University Hospital Basel, Basel, Switzerland; 4 Stoke Mandeville Hospital, Buckinghamshire NHS Trust, Oxford Thames Valley, Aylesbury, UK; 5 Medoc Swiss GMBH, Healthcare management, Basel, Switzerland; 6 School of Biomedical Sciences, The University of Queensland, St Lucia, Brisbane, 4072 Australia; 7 Department of Gastroenterology and Hepatology, Medical University Clinic, Cantonal Hospital Baselland, Liestal, Switzerland; 8 Division of General Surgery, Dalhousie University, Nova scotia, Halifax, Canada; 9 Department of Surgery, Brody School of Medicine, East Carolina University, Greenville, NC USA; 10 Faculty of medicine, University of Basel, Basel, Switzerland

**Keywords:** Crohn’s disease, Bowel resection, Kono-S anastomosis, Functional end to end anastomosis

## Abstract

**Purpose:**

Kono-S anastomosis, an antimesenteric, functional, end-to-end handsewn anastomosis, was introduced in 2011. The aim of this meta-analysis is to evaluate the safety and effectivity of the Kono-S technique.

**Methods:**

A comprehensive search of MEDLINE (PubMed), Embase (Elsevier), Scopus (Elsevier), and Cochrane Central (Ovid) from inception to August 24th, 2023, was conducted. Studies reporting outcomes of adults with Crohn’s disease undergoing ileocolic resection with subsequent Kono-S anastomosis were included. PRISMA and Cochrane guidelines were used to screen, extract and synthesize data. Primary outcomes assessed were endoscopic, surgical and clinical recurrence rates, as well as complication rates. Data were pooled using random-effects models, and heterogeneity was assessed with I² statistics. ROBINS-I and ROB2 tools were used for quality assessment.

**Results:**

12 studies involving 820 patients met the eligibility criteria. A pooled mean follow-up time of 22.8 months (95% CI: 15.8, 29.9; I^2^ = 99.8%) was completed in 98.3% of patients. Pooled endoscopic recurrence was reported in 24.1% of patients (95% CI: 9.4, 49.3; I^2^ = 93.43%), pooled surgical recurrence in 3.9% of patients (95% CI: 2.2, 6.9; I^2^ = 25.97%), and pooled clinical recurrence in 26.8% of patients (95% CI: 14, 45.1; I^2^ = 84.87%). The pooled complication rate was 33.7%. The most common complications were infection (11.5%) and ileus (10.9%). Pooled anastomosis leakage rate was 2.9%.

**Conclusions:**

Despite limited and heterogenous data, patients undergoing Kono-S anastomosis had low rates of surgical recurrence and anastomotic leakage with moderate rates of endoscopic recurrence, clinical recurrence and complications rate.

**Supplementary Information:**

The online version contains supplementary material available at 10.1007/s00423-024-03412-x.

## Introduction

Crohn’s disease (CD) is a relapsing inflammatory condition which can affect any portion of the digestive tract, leading to long term transmural inflammation with structural bowel damage and complications such as abscesses, fistulas, and strictures [1]. In addition to the economic burden CD imposes on healthcare, with annual costs reaching €30 billion in the United States and Europe, the physical, emotional, and social consequences of the disease have shown to negatively affect patients’ quality of life [2].

When Crohn and his colleagues first described regional ileitis in 1932, surgical resection was the only effective treatment available [3, 4], but the development of safe and efficacious immunosuppressors and biological therapies over the past three decades has widened the range of medical therapies [5]. Despite those advancements in medical therapy, the cumulative risk of surgery 10 years after diagnosis of Crohn’s disease remains 46.6% [6]. However, studies have shown that endoscopic postoperative recurrence can be detected in up to 70% of CD patients as early as 1 year after intestinal resection [7] and rates of re-operation reach 20–44% at 10 years and 46–55% at 20 years [8]. From a surgical perspective, the question arises of whether alteration in the surgical method could result in decreased reoperative rates. Several theories have proposed that factors including the anastomotic technique, mesenteric involvement, lumen size, anastomotic orientation (end-to-end versus side-to-side), anastomotic method (handsewn versus stapled) and rate of anastomotic insufficiency, plays a pivotal role in postoperative disease recurrence [9]. However, there is currently no consensus on the superiority of a specific anastomotic technique [10].

Kono-S anastomosis, an antimesenteric, functional, end-to-end handsewn anastomosis, was introduced in 2011 to reduce anastomotic leakage and postoperative recurrence of CD [11]. The surgical technique consists of transecting the intestine using a linear staple cutter such that the mesenteric side is in the center of the stump, at a 90° angle to the mesentery. Reinforcing and connecting both ends of the stump creates a supporting column and helps maintain the orientation and large lumen diameter of the anastomosis. Longitudinal enterotomies are performed on the antimesenteric aspect followed by creation of the anastomosis transversely in a handsewn fashion with single-layer running sutures resulting in a large anastomosis [11]. This innovative concept excludes the mesentery from the anastomosis site while maintaining its vascularization and innervation.

The aim of this systematic review and meta-analysis is to evaluate the safety of the Kono-S anastomosis technique and assess the postoperative clinical, endoscopic, and surgical recurrence rates.

## Methods

### Search strategy and data sources

A comprehensive search of MEDLINE (PubMed), Embase (Elsevier), Scopus (Elsevier), and Cochrane Central (Ovid). databases from inception to August 24th, 2023, was conducted. The search strategy, designed and conducted by a medical reference librarian, involved keywords and controlled vocabulary for concepts including “Kono-S, anastomosis”, “functional end to end”, “Crohn’s disease”, “inflammatory bowel disease”, “enteritis regionalis” and “morbus Crohn”. The review was registered prospectively with PROSPERO (CRD42023475406). Database results were uploaded into Covidence review software where deduplication took place. In accordance with Cochrane systematic review guidelines, two reviewers (MM and DV) screened titles, abstracts and full texts based on the eligibility criteria below and conflicts were resolved by an independent third reviewer (BS).

### Eligibility criteria and quality assessment

Eligible studies must have met all the following inclusion criteria: (1) participants older than 18 years with Crohn’s disease; (2) participants undergoing bowel resection and subsequently Kono-S anastomosis technique; (3) studies reporting primary outcomes of clinical, surgical or endoscopic recurrence, complications/adverse events, or anastomosis leakage following the procedure. Randomized control trials, prospective and retrospective cohort studies, case series, abstracts and poster presentations were included. The methodological quality of each study was independently evaluated by two authors using the ROBINS-I tool for non-randomized studies and ROB2 tool for randomized studies [12, 13].

### Statistical analysis

For single-arm analyses, means of continuous variables and rates of binary variables were pooled using the random-effects model, generic inverse variance method of Der Simonian, Laird [14]. Proportions underwent logit transformation prior to meta-analysis. For two-arm analyses, pooled means and proportions were analyzed using an inverse variance method for continuous data and the Mantel-Haenszel method for dichotomous data. The weight of each study was assigned based on its variance. The heterogeneity of effect size estimates across the studies was quantified using the Q statistic and the I^2^ index (*P* < 0.10 was considered significant). A value of I^2^ of 0–25% indicates minimal heterogeneity, 26–50% moderate heterogeneity, and 51–100% substantial heterogeneity. Furthermore, a leave-one-out sensitivity analysis was conducted to assess each study’s influence on the pooled estimate by omitting one study at a time and recalculating the combined estimates for the remaining studies. Data analysis was performed using Open Meta analyst software (CEBM, Brown University, Providence, Rhode Island, USA) for single arm analyses and RevMan software version 5.4 (Review Manager (RevMan) [Computer program] The Cochrane Collaboration, 2020, Copenhagen, Denmark) for two-arm analyses. If mean or standard deviation (SD) was unavailable, the median was converted to mean and the range, interquartile range or confidence intervals were converted to SD using the formulas from the Cochrane Handbook for Systematic Reviews of Interventions [15].

### Endpoints

Endoscopic recurrence was reported as Rutgeert Score ≥ i2 and mean score ranged from i0–i4 [7]. Clinical recurrence was defined as patient-reported recurrence of symptoms or Crohn’s Disease Activity Index (CDAI) > 200. Surgical recurrence was defined as reoperation with resection for recurrence of disease. Other endpoints included anastomotic leakage or insufficiency, defined as radiographic evidence of a leak from the anastomosis and included both symptomatic and asymptomatic cases. Anastomotic reintervention was defined as short term postoperative reoperation due to anastomotic leakage or insufficiency. Ileus was defined as the presence of obstructive or paralytic postoperative ileus. Infection, explained as pooled rates of wound infection, intraabdominal infection, and intraabdominal abscess was also analyzed. Biologic use preoperatively and postoperatively was also collected and analyzed.

## Results

### Study selection

The initial search yielded 157 potentially relevant articles from which 12 unique studies involving 820 patients met the eligibility criteria [11, 16–26]. The details of the study selection process and PRISMA (Preferred Reporting Items for Systematic Reviews and Meta Analyses) flow diagram are depicted in Supplementary Fig. 1.

### Risk of bias

Results of the quality assessment of all included studies are shown in Supplementary Fig. 2. In ROBINS-1, 11 studies were judged to have moderate risk of bias [11, 17–23, 25, 26], while one study was judged to have serious risk of bias [16]. In ROB2, one study was judged to have some concern for overall risk of bias [24].

### Baseline and procedural characteristics

The baseline characteristics of the included studies are comprehensively described in Table 1. 820 patients underwent 822 Kono-S anastomoses. The pooled proportion of female patients was 41.9% (*n* = 756; 95% CI: 33.5, 50.8; I^2^ = 77.9%) [11, 17–26]. The mean age of participants was 33.9 years (*n* = 756; 95% CI: 30.1, 37.7; I^2^ = 97.5%) [11, 17–26], and the pooled mean BMI was 21.97 kg/m^2^ (*n* = 574; 95% CI: 19.5, 24.5; I^2^ = 98.0%) [17, 19–22, 26]. The pooled proportion of active smokers was 21.9% (*n* = 701; 95% CI: 15.7, 29.6; I^2^ = 74.5%) [11, 17, 19–24, 26]. The pooled proportion of patients that had previous abdominal surgery was 33.9% (*n* = 512; 95% CI: 23.6, 46; I^2^ = 79.7%) [11, 17–19, 21–24]. Procedural characteristics of patients undergoing the Kono-S procedure are shown in Table 2. The total mean operative time was 215.0 min (*n* = 665; 95% CI: 138.8, 291.2; I^2^ = 99.8%) [17–22, 24–26], and the mean hospital stay was 9.3 days (*n* = 548; 95% CI: 7.2, 11.5; I^2^ = 97.4%) [17–22, 24, 25]. In the pooled proportions of surgical approach, 35.2% of procedures were open surgeries (*n* = 570; 95% CI: 22.6, 50.4; I^2^ = 87.1%) [17–25], and 64.7% were laparoscopic surgeries (*n* = 570; 95% CI: 50.6, 76.6; I^2^ = 85.5%) [17–25], while 5.6% of surgeries were converted from laparoscopic to open (*n* = 168; 95% CI: 2.7, 11.3; I^2^ = 0%) [17, 18, 22, 23, 25]. In the pooled proportions of type of anastomosis, 88.3% were small-to-large bowel (*n* = 743; 95% CI: 72.7, 95.6; I^2^ = 93.6%) [11, 17–23, 25, 26], 11.0% were small-to-small bowel (*n* = 743; 95% CI: 4.3, 25.3; I^2^ = 92.9%) [11, 17–25], and 3.1% were large-to-large bowel anastomoses (*n* = 743; 95% CI: 1.7, 5.6; I^2^ = 14.9%) [11, 17–25]. The pooled proportion of patients that completed follow up was 98.3% (*n* = 756; 95% CI: 94.9, 99.5; I^2^ = 59.2%) with a pooled mean follow-up time of 22.8 months (*n* = 758; 95% CI: 15.8, 29.9; I^2^ = 99.8%) [11, 17–26]. Pooled preoperative biologic use was 50.5% (*n* = 657; 95% CI: 36.2, 64.8; I^2^ = 89.0%) [17–21, 23–26] and postoperative biologic use was 49.8% (*n* = 411; 95% CI: 41.4, 58.1; I^2^ = 58.7%) [11, 17, 18, 20, 22–24, 26]. No difference was found between the two groups when comparing biologic use preoperatively and postoperatively (OR = 1.26; 95% CI: 0.46, 3.46; I^2^ = 84%, *p* = 0.65) [17, 18, 20, 23, 24, 26].

**Table 1:** Baseline characteristics of included studies and patients

Study	Country	Type of Study	No. of Participants	No. of Procedures	Gender Female - *N* (%)	Mean Age ± SD	Mean Current BMI ± SD	Smokers - *N* (%)	Previous Abdominal Surgery - *N* (%)
Adamou [16]	Germany	Retrospective	64	64	NR ^a^	NR	NR	NR	NR
Alibert [17]	France	Prospective	61	61	35 (57%)	37 ± 14.1	21.9 ± 3.5	16 (26%)	12 (20%)
Eto [18]	Japan	Retrospective	2	2	1 (50%)	25.5 ± 0.3	NR	NR	0 (0%)
Fichera [19]	USA	Retrospective	262	262	122 (47%)	28.8 ± 14.9	24.5 ± 5.6	44 (17%)	135 (52%)
Holubar [20]	USA	Retrospective	74	74	38 (51%)	38.2 ± 16.3	25.1 ± 5.6	12 (16%)	NR
Horisberger [21]	Switzerland	Prospective	30	30	14 (47%)	32 ± 14.5	23 ± 5.9	8 (27%)	6 (20%)
Katsuno [22]	Japan	Prospective	30	32	8 (27%)	34 ± 6.3	18.6 ± 3.0	9 (30%)	12 (40%)
Kelm [23]	Germany	Retrospective	22	22	8 (36%)	37.4 ± 10.5	24.3 ± NR	5 (23%)	6 (27%)
Kono [11]	Japan	Retrospective	69	69	12 (17%)	31 ± 10.8	NR	25 (36%)	21 (30%)
Luglio [24]	Italy	Randomized Control Trial	36	36	18 (50%)	34 ± 6.3	NR	11 (31%)	19 (53%)
Seyfried [25]	Germany	Retrospective	53	53	32 (60%)	37 ± 11	NR	NR	NR
Shimada [26]	Japan	Retrospective	117	117	33 (28%)	39 ± 11.9	18.9 ± 2.5	7 (6%)	NR

a NR = Not Reported

**Table 2:** Procedural characteristic and biologic use

Study	No. of Procedures	Mean Total Operative Time (Min) ± SD	Mean Hospital Stay (Days) ± SD	Surgical Approach				Type of Anastomosis				Follow-Up			Biologics Treatment	
				Open *N* (%)	Laparoscopic *N* (%)	Converted from Lap to open *N* (%)	Small Bowel to Large Bowel (%)		Small Bowel to Small Bowel (%)	Large Bowel to Large Bowel (%)	*N* of Follow Up (%)		Mean Months ± SD	Preoperative *N* (%)		Postoperative *N* (%)
Adamou [16]	64	NR ^a^	NR	NR	NR	NR	NR		NR	NR	NR		NR ± NR	NR		NR
Alibert [17]	61	140 ± 54.8	6 ± 2.2	5 (8%)	56 (92%)	0 (0%)	61 (100%)		0 (0%)	0 (0%)	61 (100%)		6.5 ± 1.68	57 (93%)		30 (49%)
Eto [18]	2	358 ± 0.5	24.5 ± 1.8	2 (100%)	0 (0%)	0 (0%)	2 (100%)		0 (0%)	0 (0%)	2 (100%)		21.5 ± 1.25	1 (50%)		0 (0%)
Fichera [19]	262	274.4 ± 103.3	5.7 ± 3.9	53 (20%)	204 (78%)	NR	231 (88%)		31 (12%)	0 (0%)	262 (100%)		49.4 ± 17.6	150 (57.3%)		NR
Holubar [20]	74	170 ± 107.8	4 ± 4.3	30 (41%)	44 (59%)	3 (6.8%)	74 (100%)		0 (0%)	0 (0%)	74 (100%)		5 ± 21.5	46 (62%)		50 (68%)
Horisberger [21]	30	256 ± 54.8	9 ± 3.7	10 (33%)	20 (67%)	NR	30 (100%)		0 (0%)	0 (0%)	30 (100%)		10 ± 5.56	19 (63%)		NR
Katsuno [22]	32	199 ± 54.8	15 ± 14.8	23 (77%)	8 (27%)	1 (3%)	24 (75%)		6 (19%)	2 (6%)	30 (100%)		35 ± 13.25	NR		19 (63%)
Kelm [23]	22	161 ± NR	8.1 ± NR	10 (45%)	12 (55%)	1 (5%)	22 (100%)		0 (0%)	0 (0%)	22 (100%)		8.8 ± 2.5	5 (23%)		8 (36%)
Kono [11]	69	NR	NR	NR	NR	NR	46 (51%)		40 (45%)	4 (4%)	69 (100%)		42 ± 18.75	NR		29 (42%)
Luglio [24]	36	165 ± 42	7 ± 3.0	17 (47%)	19 (53%)	NR	NR		NR	NR	36 (100%)		24 ± 0	14 (39%)		14 (39%)
Seyfried [25]	53	157 ± 62.8	8 ± 6.3	10 (19%)	42 (79%)	4 (8%)	51 (94%)		3 (6%)	0 (0%)	46 (87%)		12 ± 4.75	14 (26%)		NR
Shimada [26]	117	215 ± 71.9	NR	NR	NR	NR	42 (36%)		69 (59%)	5 (4%)	117 (100%)		38 ± 23.70	36 (31%)		58 (50%)

a NR = Not Reported

### Outcomes of kono-S procedure

**Fig. 1:**
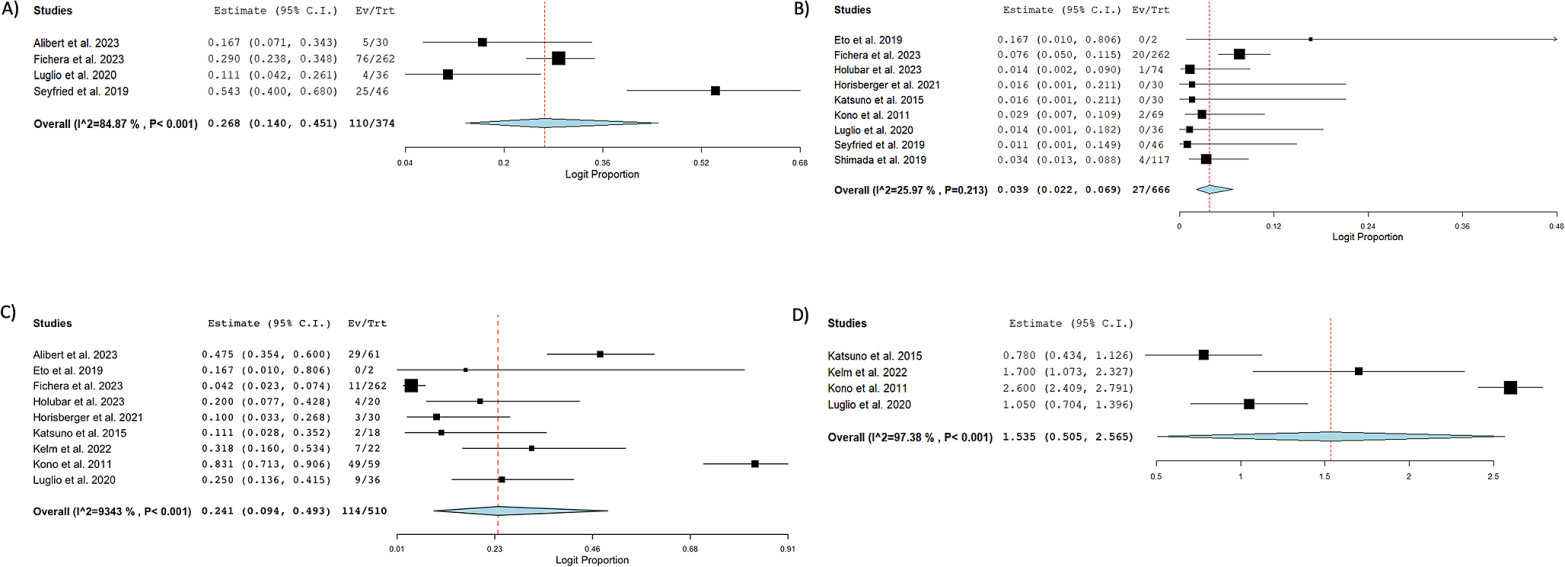
Forest plots of recurrence. **A**) Pooled rate of Clinical Recurrence **B**) Pooled rate of Surgical Recurrence **C**) Pooled rate of Endoscopic Recurrence **D**) Pooled mean Rutgeert Score

**Fig. 2:**
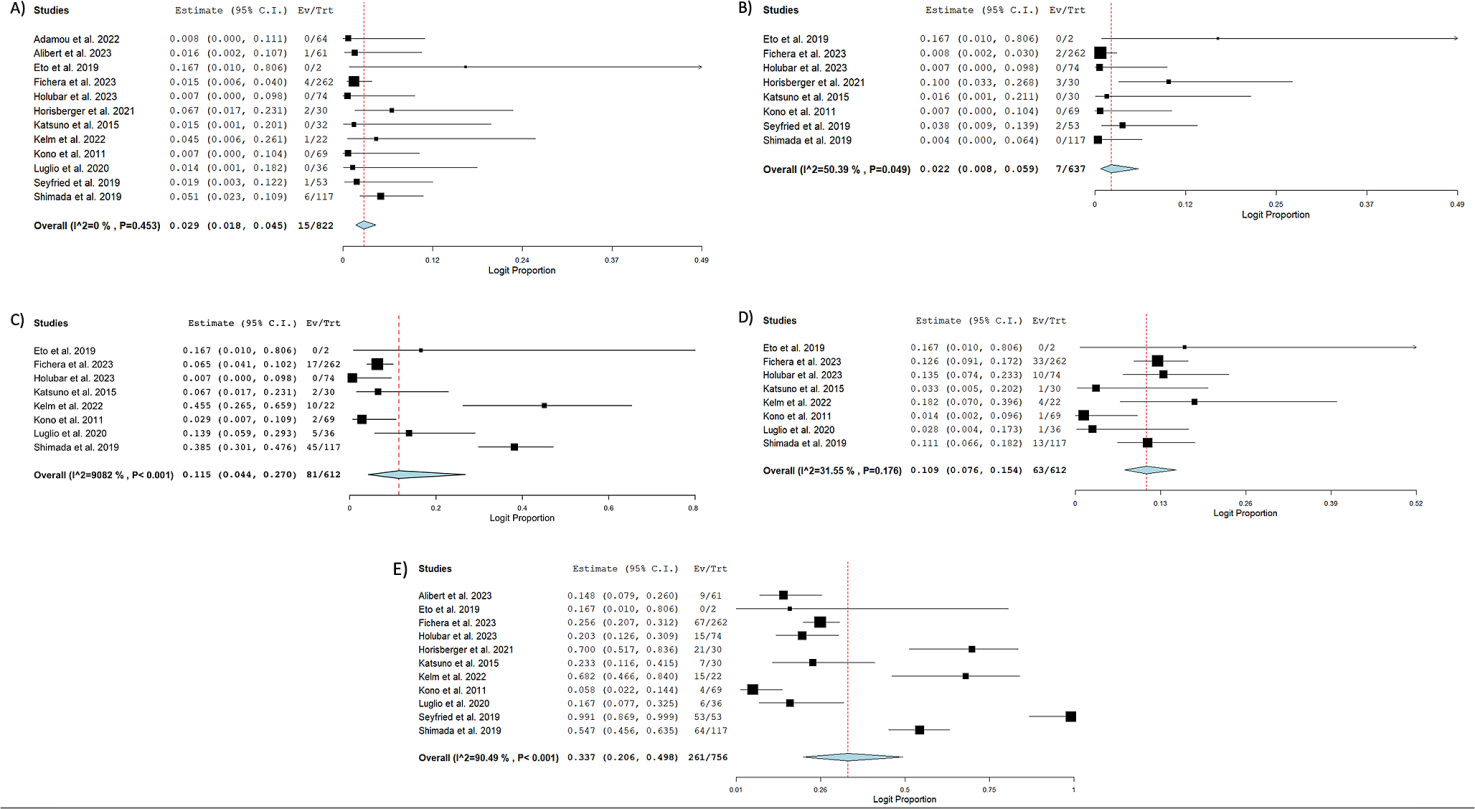
Forest plots of complications, anastomosis leakage and reintervention. **A**) Pooled rate of Anastomosis Leakage **B**) Pooled rate of Reintervention **C**) Pooled rate of Infection **D**) Pooled rate of Ileus **E**) Pooled total Rate of Complications

Outcomes of the Kono-S procedure are outlined in Table 3. The pooled clinical recurrence was 26.8% (*n* = 374; 95% CI: 14, 45.1; I^2^ = 84.9%) [17, 19, 24, 25]. The pooled surgical recurrence rate was 3.9% (*n* = 666; 95% CI: 2.2, 6.9; I^2^ = 25.97%) [11, 18–22, 24–26]. The pooled endoscopic recurrence rate was 24.1% (*n* = 510; 95% CI: 9.4, 49.3; I^2^ = 93.43%) [11, 17–24]. The pooled mean Rutgeert score measured in 157 patients at follow up was 1.54 (95% CI: 0.51, 2.57; I^2^ = 97.4%) [11, 22–24]. Recurrence rates are depicted in Fig. 1. The pooled anastomosis leakage rate was 2.9% (*n* = 822; 95% CI: 1.8, 4.5; I^2^ = 0%) [11, 16–26]. The pooled rate of patients requiring reoperation for complications or anastomotic leakage was 2.2% (*n* = 637; 95% CI: 0.8, 5.9; I^2^ = 50.4%) [11, 18–22, 25, 26]. The most common complication was infection, with a pooled rate of 11.5% (*n* = 822; 95% CI: 4.4, 27.0; I^2^ = 90.8%) followed by ileus with a pooled rate of 10.9% of participants (*n* = 612; 95% CI: 7.6, 15.4; I^2^ = 31.6%) [11, 18–20, 22–24, 26]. 261 total complications were recorded with a pooled proportion of 33.7% (*n* = 756; 95% CI: 20.6, 49.8; I^2^ = 90.5%) [11, 17–26]. Complication, anastomotic leakage, and reintervention rates are comprehensively shown in Fig. 2.

**Table 3:** Outcomes of the Kono-S anastomosis

Study	Clinical Recurrence *N* (%)	Surgical Recurrence *N* (%)	Endoscopic Recurrence		Anastomosis Leakage *N* (%)	Patients requiring Reintervention *N* (%)	Complications			
			*N* of recurrence (%)	Mean Rutgeert score ± SD			Infection	Ileus	Other	Total Complications (*N*)
Adamou [16]	NR ^a^	NR	NR	NR	0 (0%)	NR	NR	NR	NR	NR
Alibert [17]	5 (17%)	NR	29 (48%)	NR	1 (2%)	0 (0%)	NR	NR	NR	9
Eto [18]	NR	0 (0%)	0 (0%)	NR	0 (0%)	0 (0%)	0	0	NR	0
Fichera [19]	76 (29%)	20 (8%)	11 (4%)	NR	4 (1.5%)	2 (1%)	17	33	13	67
Holubar [20]	NR	1 (1%)	4 (20%)	NR	0 (0%)	0 (0%)	0	10	5	15
Horisberger [21]	NR	0 (0%)	3 (10%)	NR	2 (7%)	3 (10%)	NR	NR	18	21
Katsuno [22]	NR	0 (0%)	2 (11%)	0.78 ± 0.75	0 (0%)	0 (0%)	2	1	0	7
Kelm [23]	NR	NR	7 (32%)	1.7 ± 1.5	1 (5%)	NR	10	4	0	15
Kono [11]	NR	2 (3%)	49 (83%)	2.6 ± 0.75	0 (0%)	0 (0%)	2	1	0	4
Luglio [24]	4 (11%)	0 (0%)	9 (25%)	1.05 ± 1.06	0 (0%)	NR	5	1	0	6
Seyfried [25]	25 (54%)	0 (0%)	NR	NR	1 (2%)	2 (4%)	NR	NR	NR	53
Shimada [26]	NR	4 (3%)	NR	NR	6 (5%)	0 (0%)	45	13	0	64

a NR = Not Reported

## Discussion

Initial Crohn’s disease (CD) recurrence has been shown to occur proximal to the surgical anastomosis [27, 28], with histopathological inflammatory changes detected as early as 1 week postoperatively [29]. As a result, from a surgical perspective there has been an increasing focus on the choice of resection and anastomotic techniques. Our study demonstrated a postoperative endoscopic recurrence rate of 24.1%, which is lower than that reported in a study assessing patients receiving a conventional anastomosis, with a recurrence rate of 42.6% [28]. This indicates that the Kono-S anastomosis may have a protective role in endoscopic recurrence and CD progression at the anastomotic site. However, this observed difference is debatable due to a lack of comparative studies and long term follow up in our population and the remaining literature. Our study’s pooled follow-up period was 22.8 months; however, the heterogeneity of follow-up in included studies was considerable at 99% due to varied monitoring lengths and the lack of standardized follow-up protocols. In addition, there is a possibility that our recurrence rates were underestimated since some studies reported recurrence as Rutgeerts score ≥ i2 while others reported is as > i2. Surgical recurrence remains important in ileocolic disease with estimated rates of recurrence requiring re-operation of 11–32% at 5 years and 46–55% at 20 years [8]. A randomized controlled trial comparing handsewn, and stapled end-to-end anastomoses in 63 patients reported surgical recurrence rates of 7.5% and 5% at 24 months, respectively [30]. Additionally, a prospective study in 30 patients indicated that side-to-side anastomoses resulted in recurrence rates ranging from 0 to 4% over the same follow-up period [31]. When compared to our recurrence rate of 3.9% at 22.8 months, the observable differences are minimal, and conclusions are difficult to draw due to the small sample size. These findings underscore the necessity for large-scale, comparative randomized trials to further investigate these outcomes. Our study also demonstrated a pooled rate of clinical recurrence of 26.8% in 374 patients at follow up, but results were deemed to have high heterogeneity. In population-based studies, the clinical recurrence rate ranged from 28 to 45% and 36–61% at 5 and 10 years, respectively [32]. However, it is difficult to judge the clinical recurrence rate in our study due to a low sample size and unstandardized definitions of clinical recurrence across studies.

It is important to consider postoperative use of biologics and other immunomodulators as prophylaxis for recurrence. Our study demonstrated that there was no difference between biologic administration rates pre- and post-operatively, however, our findings are limited by high heterogeneity. Although this could indicate that recurrence rates observed were mainly influenced by Kono-S technique, the effect of other pertinent risk factors including active smoking or previous abdominal surgery could not be isolated in our study.

The total rate of complications in our meta-analysis was 33.7%. In contrast, other studies showed a 23% complication rate in stapled side-to-side anastomosis and a 21–24% rate in end-to-end anastomosis [10, 33]. However, several comparative cohort and randomized studies showed either no difference or significantly reduced complication rates in Kono-S anastomosis when compared to other surgical techniques [11, 17, 21, 23, 24, 26]. Given the high heterogeneity in the data (90%) and large confidence intervals, a larger sample size and more studies are required to get a more representative rate.

Regarding anastomotic leak, our study reported a rate of 2.9%. Other studies examining anastomotic insufficiency have shown a 4–7% leak rate in stapled side-to-side anastomosis and a 7-8.6% rate in end-to-end anastomosis [10, 33, 34]. This is an observable difference that has been corroborated by a randomized trial and comparative cohort studies [11, 24, 26]. However, it is difficult to determine clinical significance due to the low number of patients in the current study and the rare nature of this complication; thus, large, multi-center randomized trials are required to elucidate any potential difference, although they may not be feasible as additional factors such as surgeon preference, increasing number of medical therapies along with decreasing rates of surgical interventions may render a randomized trial unfeasible [35]. The most common complication in our study was infection occurring in 11.5% followed by ileus in 11%. A systematic review revealed similar rates of postoperative ileus after colorectal surgery, occurring in 10% [36]. The pooled rate in our study is higher than that reported in the literature examining other anastomotic techniques, and this could be explained by the prolonged duration of the Kono-S operation and exposure to anesthesia when compared to traditional anastomotic techniques [37]. In our study, the mean duration of operation was 215 min and was observably longer than stapled side-to-side (113 min) and end-to-end anastomosis (138 min) [33]. This longer duration can be attributed to the novel nature and learning curve of the technique, which also contributes to the increased heterogeneity in our data. Regarding total hospital stay, our study reported an average of 9.3 days, with significant heterogeneity. Notably, a leave-one-out analysis with the exclusion of Eto et al., [18] yielded a result of 6.8 days, which is comparable to the literature on side-to-side and end-to-end anastomoses [10, 24, 33, 38].

Several factors related to anastomoses have been postulated to play a role in disease recurrence. First, a growing body of research has demonstrated that mesenteric organs, such as adipose tissue, lymphatics, blood vessels, and mesenteric nerves play a critical role in the etiology and progression of CD [19]. The involvement of the mesentery has largely been thought to be secondary to CD, but recent literature has demonstrated evidence of mesenteric abnormalities detected prior to any mucosal changes can subsequently predict development of CD [39]. This was further confirmed by a retrospective study showing that excision of the mesentery significantly reduced reoperation rates [40]. Even though the mesentery is preserved in the Kono-S technique, the antimesenteric lumen and the posterior supporting column that excludes the mesentery from the lumen itself could be a contributing factor to the reduction in recurrence rates, as demonstrated in the current study [19]. Second, the effect of lumen size and configuration on enteric flow has recently gained popularity. Since fecal stasis is suspected to influence mucosal healing and has been linked to disease recurrence [17, 26], ECCO Guidelines currently advocate carrying out a wide ileo-colic anastomosis [41]. The antimesenteric configuration of the Kono-S anastomosis results in a consistently wide lumen (7 cm diameter) and prevents unnecessary denervation or devascularization of the anastomotic site [11]. This supports the healing process and lessens the risk of secondary ischemia, early stenosis, colonic reflux, and fecal stasis [42]. Moreover, by stabilizing the lumen, the posterior column prevents distortion and reduces the likelihood of enteric flow impediments [11]. The Kono-S anastomosis positively benefits endoscopists by increasing the feasibility of endoscopic monitoring and intervention, as the wide lumen at the anastomotic site and the supporting column maintain a three-dimensional structure [21]. Last, there is evidence linking postoperative complications, particularly septic complications, to a higher chance of recurrence [19, 43]. The safety of the Kono-S technique, as demonstrated by the low rates of anastomotic leakage and infection in our meta-analysis, may further contribute to reduced recurrence rates.

This meta-analysis builds on the foundation laid by previously published systematic reviews [44, 45]. While acknowledging the valuable contributions of previous work, our meta-analysis addressed limitations of overlapping populations and the need for continual updates when evaluating a new surgical technique such as the Kono-S anastomosis. In addition, 11 studies were excluded due to the possibility of shared participants and six new articles that were not previously included in any systematic reviews were added. These changes enhanced generalizability, statistical power, and reliability and reduced potential bias.

Several limitations must be taken into consideration. First, the majority of included studies were retrospective in design, which removed the possibility of randomization and introduced selection bias. Second, the reproducibility of the results was reduced since most studies were conducted in a single center. Furthermore, the high heterogeneity in follow up periods between studies limits the conclusions drawn about recurrence rates in the studied population. Moreover, the occurrence of various types of anastomoses, such as small to large bowel, large to large bowel, and small to small bowel, in our investigated population introduces bias into our results. In addition, we were unable to assess the difference between emergency and ambulatory indications, which could introduce a confounding bias in the studied population. Finally, this procedure is relatively novel and requires surgical expertise. Since the ability to assess the surgeon’s learning curve and skills for Kono-S anastomosis was not possible, heterogeneity and bias in the results should be taken into account. Despite these limitations, to our knowledge, this is one of the largest and most comprehensive studies available in the literature. It demonstrates that the Kono-S anastomosis in Crohn’s patients undergoing surgical resection could be a possible alternative to other techniques.

In summary, this meta-analysis presents preliminary evidence evaluating the safety and effectivity of the Kono-S procedure in Crohn’s disease patients undergoing ileocolic resection. Despite limited data in this meta-analysis, there appears to be a relatively low rate of surgical recurrence, and a relatively moderate rate of endoscopic and clinical recurrence in patients undergoing Kono-S. Moreover, there appears to be a promising trend suggesting a moderate complication rate and a low anastomotic leakage rate. Given the results, further studies with increased sample sizes and longer follow up are required to elucidate the safety and effectivity of Kono-S technique.

### Electronic supplementary material

Below is the link to the electronic supplementary material.

Supplementary Material 1

## Data Availability

With the publication, the data set used for this meta-analysis will be shared upon request from the corresponding authors.
