# Supplementary material for: Safety and effectivity of Kono-S anastomosis in Crohn’s patients: a systematic review and Meta-analysis
Source: Langenbecks Arch Surg. 2024 Jul 22;409(1):227. doi: 10.1007/s00423-024-03412-x (PMC11263246; doi:10.1007/s00423-024-03412-x)
Supplement: Supplementary file 1 — Supplementary Material 1 [file 423_2024_3412_MOESM1_ESM.docx]

**Supplements**

**Total Supplements: 2 Figures**

- **Figure 1:** PRISMA Flow diagram of study selection
- **Figure 2:** Risk of Bias Assessment – ROBINS-I and ROB2


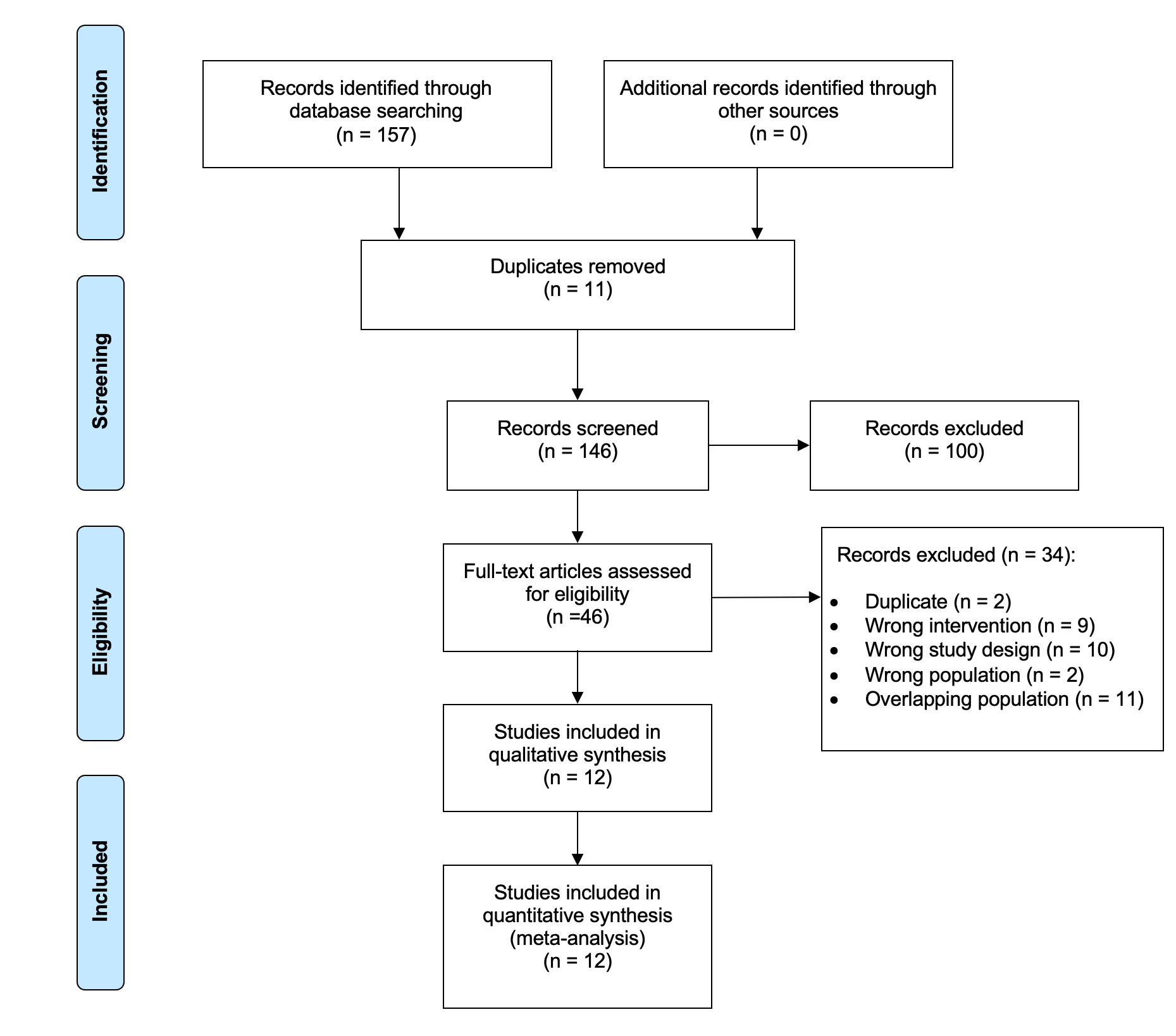


**eFigure 1: PRISMA Flow diagram of study selection**


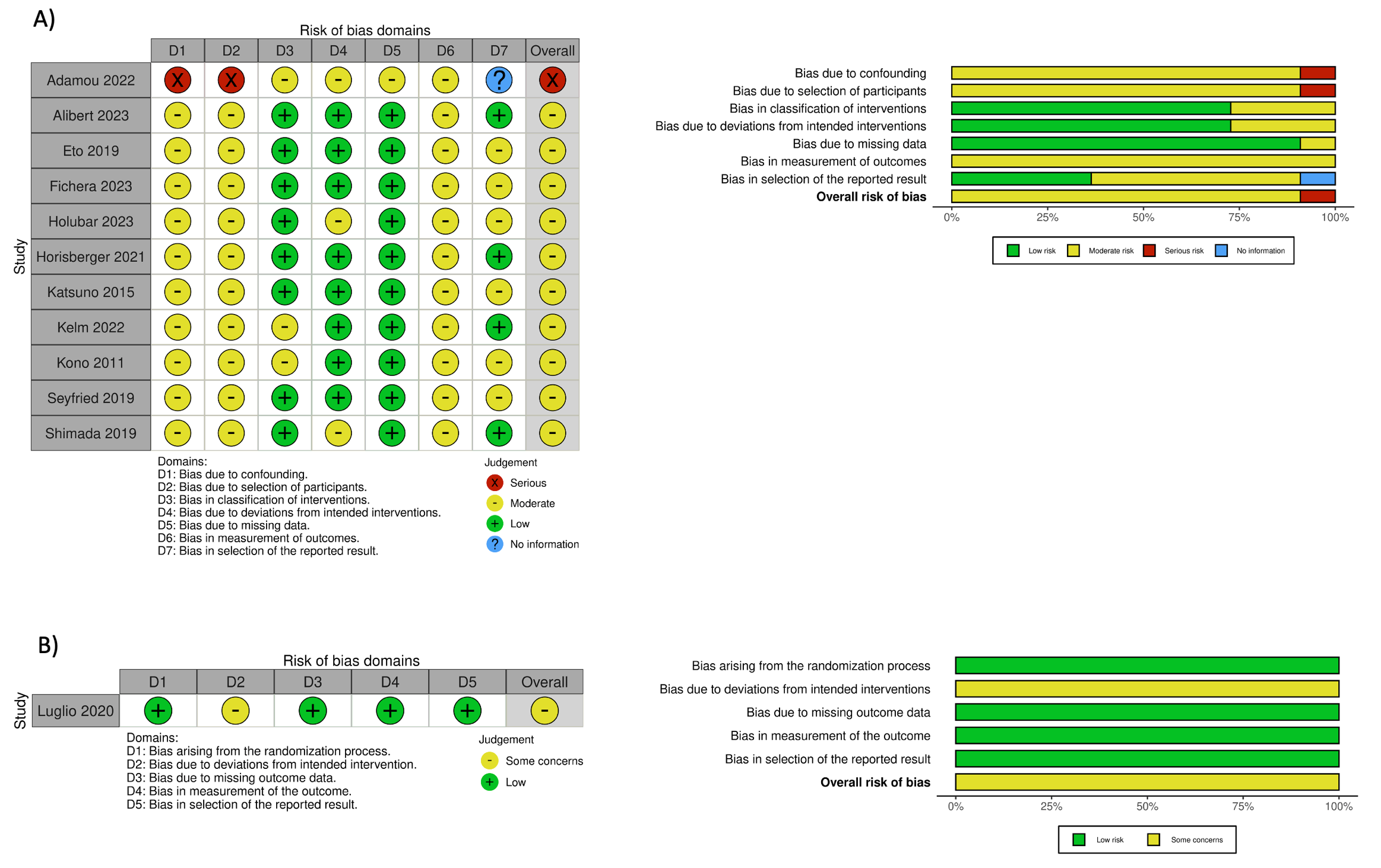


A) ROBINS-I assessment for non-randomized studies

B) ROB-2 assessment for randomized control trials

**eFigure 2: Risk of Bias Assessment – ROBINS-I and ROB2**
